# Surgical Proposition of a Slow-Growing Calvarial Exostosis in a Female Patient With a Congenital Iris Cyst of the Anterior Chamber and Mandibular Tori

**DOI:** 10.7759/cureus.56642

**Published:** 2024-03-21

**Authors:** Bita C Behaeddin, Monica M Ramos, Omar G Jarrett, Ernesto V Torres

**Affiliations:** 1 School of Medicine, St. George's University School of Medicine, New York, USA; 2 General Surgery, Sanitas Medical Center, Miami, USA; 3 School of Medicine, Ross University School of Medicine, Miami, USA

**Keywords:** osteoma, gardner's syndrome, hereditary multiple exostoses, ct scan head, skull base tumors, radiological findings, iris cyst, mandibular tori, calvarial exostosis

## Abstract

We present an unusual case of a woman in her early 50s with a slow-growing calvarial exostosis. Exostoses are bony spurs or osteomas extending outward beyond a bone's surface and may be benign or malignant. Calvarial exostoses are a less common bone tumor that can occur in the population. We present a case of a rare, slow-growing calvarial exostosis with a combination of mandibular tori and a congenital iris cyst. We discuss differentials of this exostosis and different syndromes that may cause it such as hereditary multiple exostoses and Gardner syndrome. The current article aims to spread awareness of this atypical presentation of exostoses and present our institution's surgical proposition for removing a calvarial exostosis to obtain a further histological analysis of its composition. As these masses may commonly be benign, a definitive diagnosis cannot be made through imaging alone to rule out more threatening conditions. We have addressed radiological findings and diagnostic and treatment options offered to the patient. The patient decided not to move forward with removing the mass and would continue to monitor and return should she notice any unusual or acute changes.

## Introduction

Exostoses are slow-growing, benign growths that protrude from the bone surface [1]. As a quick definition of terms, exostosis can refer to an osteoma and mandibular tori are bony outgrowths of the mandible. Depending on the region of the skeletal system where an exostosis forms, it may be asymptomatic or irritating. Asymptomatic growths undergo surgical excision mainly for cosmetic purposes. Osteomas comprise trabecular and compact bone and are typically from the outer table. CT of the head usually shows a well-depicted solitary round or oval lesion that has homogenous enhancement with a dense structure [2].

Gardner syndrome is a condition that raises suspicion due to the osteomas present in this patient's mandible as well as the skull. This condition typically presents as a variant of familial adenomatous polyposis and can include multiple osteomas and congenital ocular fundus lesions [3]. Usually, these do not require surgical treatment unless they invade other structures. Congenital iris cysts may be primary, meaning we do not know the origin, or secondary, meaning they have a known cause. They are rare; studies show 21% are cystic, of which 87% are pigment epithelial cysts. These cysts are also more common in men than females [4]. It is essential to mention that multiple exostoses are rare and may raise suspicion for conditions such as hereditary multiple osteochondromas. This condition often results from loss of function mutations in the EXT1 and EXT2 tumor suppressor genes, which have functions in heparan sulfate proteoglycan synthesis. One of the feared complications of certain exostoses is the formation of chondrosarcoma. However, 1-2% of benign exostoses become chondrosarcomas. However, this patient presented with a benign osteoma, which did not contain cartilage, and we had no documented cases of malignant transformation. Mandibular tori are benign and often asymptomatic and incidentally found on the lingual side of the mandible, with surgical resection rarely necessary due to its harmless nature. Tori is most common in males, and frequency increases with age, although this patient is female. There are higher prevalence rates in Asian, European, and Caucasian populations [5-11]. This rare case describes a woman with a bony mass growing over her left parietal skull, a mandibular tori, and a history of a congenital iris cyst. Surgical excision was necessary to confirm a diagnosis of calvarial exostosis; therefore, it could not be considered wholly benign or malignant.

Our case study is believed to be the first to investigate this clinical sign as an indicator of a syndrome that may include ophthalmologic and additional osseous findings. We are contributing this case report to the literature due to the findings of this case that may illuminate the possible pathogenesis or unique features of an existing disease or one not yet discovered.

## Case presentation

A 52-year-old female patient presented to the Sanitas Medical Center in Miami, Florida, USA, in January of 2023 with concerns about a slow-growing mass on the left side of her head. The mass was said to have appeared about two decades before her appointment. She stated that the mass had started small and had slowly grown at an unknown rate. The patient denied any pain or itching related to the mass. She only reported that she felt some mild discomfort over the area when lying her head down on her left side. She has a past medical history of mandibular tori and a congenital iris cyst of the anterior chamber of the right eye. Her previous surgical history included the complete removal of the iris cyst a couple of months before presenting to us on this visit. Family history was unremarkable, except for the patient disclosing that her daughter had been diagnosed with Eagle syndrome and had also developed mandibular tori.

**Figure 1:**
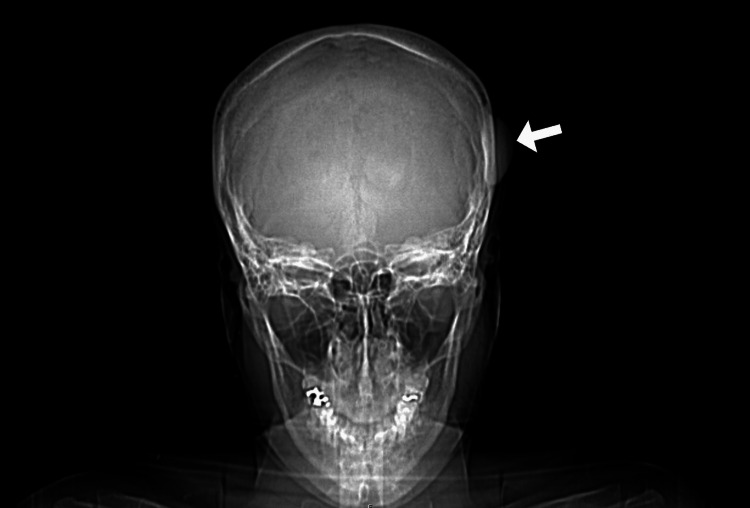
Coronal view of the skull showing a 3.71 x 1.36 x 4.2-centimeter mass on the outer table of the left temporoparietal calvarium

**Figure 2:**
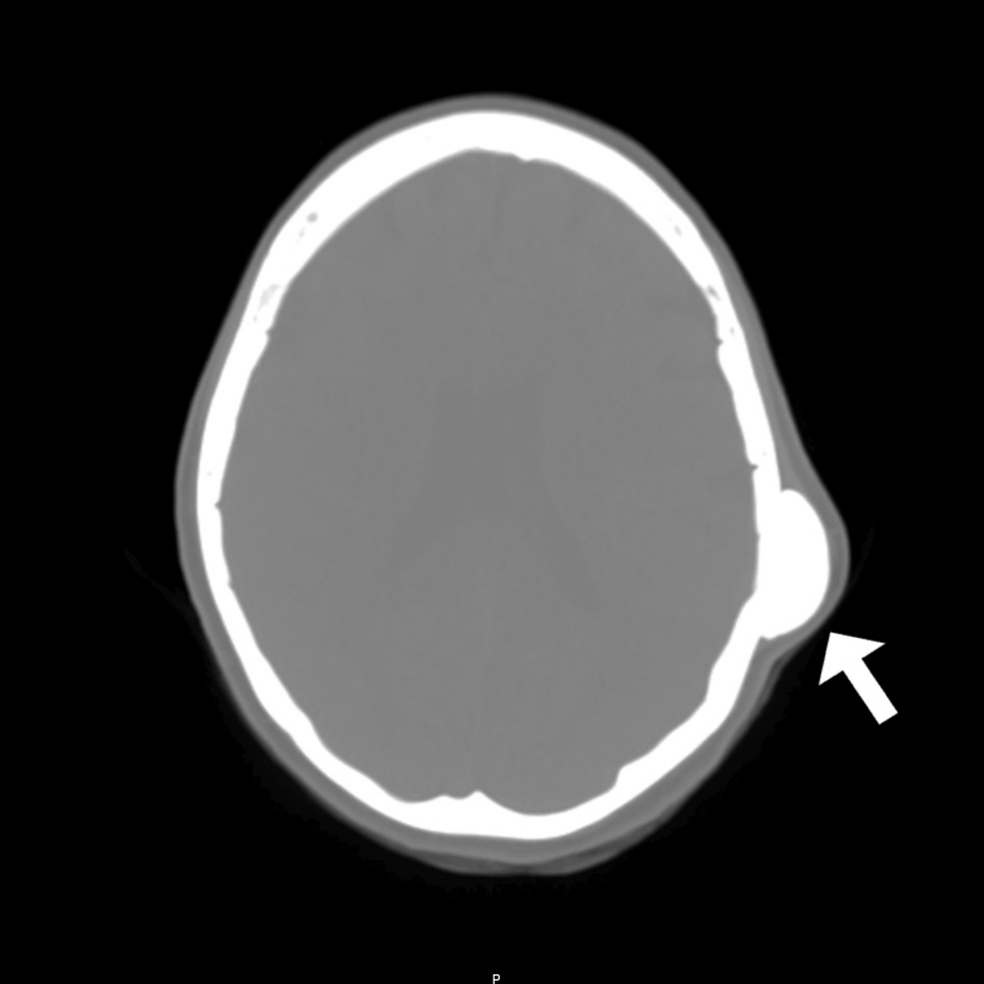
Axial view of the skull showing a 3.71 x 1.36 x 4.2-centimeter mass on the outer table of the left temporoparietal calvarium

On physical examination, a firm, immobile 3x4 cm mass was palpated on her left parietal bone. No lesions were visualized on the overlying skin. Bony outgrowths on the lingual surface of the mandible were present bilaterally, consistent with mandibular tori. There was no tenderness to the skull or the mandibular tori. CT without contrast of the skull base to the vertex was ordered and obtained and revealed an ossified, crescent-shaped, 3.71 x 1.36 x 4.2-centimeter mass on the outer table of the left temporoparietal calvarium as shown in Figure 1 and Figure 2. The findings upon imaging were consistent with a calvarial exostosis.

The patient was given the option of undergoing surgery for the removal of the mass but had denied any surgical intervention at the time. The mass was identified in the outer table of the left temporoparietal calvarium without intracranial extension, leading us to presume it to be a benign calvarial exostosis. The patient was offered the treatment option of removal via a linear incision across the scalp and excision by cutting the base of the mass. Pathology and histological confirmation of the mass were not obtained because the patient had not undergone any intervention for the mass. The patient agreed to follow up as needed if any changes or concerns arose. If such changes or concerns presented themselves, another CT head without contrast would be ordered to document further mass changes. Neurosurgery referral would also be made should the mass begin to demonstrate intracranial extension or neurological symptoms.

## Discussion

Calvarial skull lesions can vary from benign to malignant. Generally, these growths are asymptomatic, and surgical excision is mainly undergone for cosmetic purposes. Exostoses etiology is poorly understood at this time, although genetic factors may play a role, as in this patient whose daughter presented with similar mandibular tori. As previously mentioned, mandibular tori are exostoses located on the mandible's lingual side, with surgical resection rarely being necessary due to its benign nature [4]. Further research is required into her condition, as this patient is female and tori are more common in males. Multiple osteomas in the skull would promote workup for Gardner syndrome, a possible phenotype of familial adenomatous polyposis. It is worth noting that a distinguishing feature of Gardner syndrome is congenital ocular lesions. The ocular lesions are derived from the retinal epithelium in Gardner syndrome; the patient described a congenital iris cyst [2]. Although this patient presented with osteomas of the skull and mandible, she had no history of colonic polyps.

Another possible condition is hereditary multiple exostoses, which can present with the progression of these exostoses to chondrosarcomas, although this is rare [5,11]. However, this patient did not undergo any pathological testing, and it is impossible to rule it in or out.

To date, this patient has not displayed any obvious lymphadenopathy of the head and neck, which is common in malignant tumors [10]. This supports the evidence that the calvarial exostosis present in this patient's case is benign.

The patient's daughter has a medical history of Eagle syndrome, which is a disease that is described as the lengthening of the styloid process and infringement of the stylohyoid ligament. The cause is deemed to be inflammation or irritation, which leads to ossification. However, the disease may occur without irritation or trauma. There are two types: the classic, which occurs most commonly after a trauma to the throat, and the carotid type, which occurs due to carotid artery constriction. As our patient denies any history involved with her styloid process or stylohyoid ligament nor any relevant history of trauma to the throat or carotid artery constriction, this disease process is unlikely [12]. Information about a relationship between this patient's exostoses of mandibular tori and the calvarium and Eagle Syndrome has yet to be found.

As our patient will not be undergoing surgery for removal and further analysis, she will continue to be monitored closely through additional imaging and follow-up appointments. Genetic testing of this patient is necessary to make an accurate diagnosis. The literature does not report a patient with concurrent calvarial exostosis, congenital iris cyst, and mandibular tori.

## Conclusions

Exostoses can be called bony spurs or osteomas but are bone growths extending beyond a bone's surface. Calvarial skull masses may range from benign to malignant. Evidence from the head CT showed a mass on the outer table of the left temporoparietal calvarium with no intracranial extension, along with it being slow-growing over two decades, leading to the presumption that it was benign. The patient, however, refused removal treatment. This patient's previous medical history of a congenital iris cyst and mandibular tori warrant a possible investigation. Although these masses may commonly be benign, pathology and genetic testing may be necessary to determine any underlying threatening conditions.
